# High output power low temperature polysilicon thin-film transistor boost converters for large-area sensor and actuator applications

**DOI:** 10.1038/s41528-026-00536-6

**Published:** 2026-01-27

**Authors:** Mauricio Velazquez Lopez, Nikolas Papadopoulos, Paoline Coulson, Bjorn Vandecasteele, Kris Myny

**Affiliations:** 1 EPIC, Large Area Thin-film Transistor Electronics, imec, Leuven, Belgium; 2 ES&S-ESAT, KU Leuven, Diepenbeek, Belgium; 3 KU Leuven, Nerf, imec, Leuven, Belgium; 4 CMST, imec and Ghent University, Technologiepark, Gent, Belgium

**Keywords:** Electrical and electronic engineering, Electronics, photonics and device physics, Nanoscience and technology

## Abstract

Large-area electronic sensor and actuator arrays are suitable systems for thin-film transistor (TFT) technology with numerous applications from consumer electronics to healthcare. Considerable effort is being spent to make these arrays a reality. However, research on the power delivery circuits that supply these arrays has remained largely unexplored. This work delves into the design trade-offs and characterization of high output power boost converters in low-temperature polysilicon (LTPS) technology. The proposed boost converters deliver 0.62–2.17 W of output power, orders of magnitude above prior TFT solutions, with efficiencies ranging from 47 to 69.5%. These boost converters enable the realization of large-area sensor and actuator arrays and set the foundation for future research in this area.

## Introduction

**Fig. 1:**
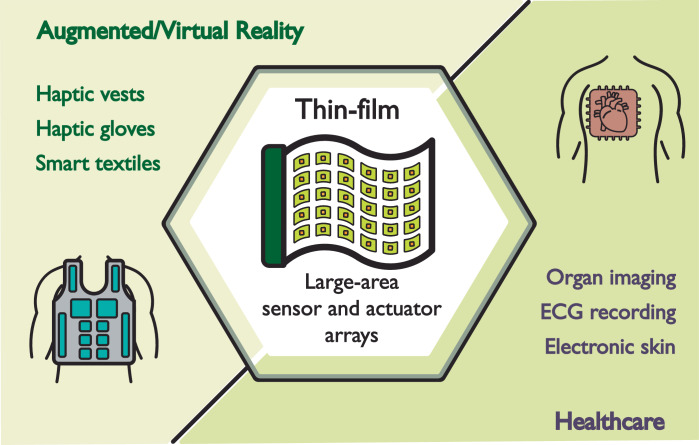
Applications requiring large area coverage by sensors and actuators, such as haptic gloves, haptic vest, smart textiles, organ imaging, electrocardiogram (ECG) recording, and electronic skin. The following icons were used and modified for the creation of this figure: “Heart” by N.Style for the body, “Heart” by Minh Do for the heart, “patch” by Puspa Kusuma for the patch, “vest” by Singlar for the vest and “Flag” by Larea for the thin-film outline; all from thenounproject.com.

Thin-film transistors (TFTs) are a natural fit for applications that require a significant number of sensors and actuators distributed over large surface areas, some of which are shown in Fig. 1. The inherent low cost per area that results from the TFTs’ simpler manufacturing processes makes large area coverage affordable compared to their silicon CMOS counterparts. Furthermore, TFT technology can also be manufactured on flexible substrates, which enables the final circuits to better conform to surfaces with complex topologies such as the human body^1^. Thanks to these advantages, the potential use of TFTs for several large-area systems is already being investigated^2–10^.

In healthcare applications, large-area arrays need to conform to the patient’s skin while covering it with numerous sensors in order to monitor a specific organ or set of vital signs. For example, ultrasound transducers are currently being used for organ monitoring applications^2,3^. These transducers require relatively high voltages to be operated and must be present in the thousands to achieve the beam steering effect necessary for organ imaging^4^. Other notable health-related applications include strain sensor arrays for pulse monitoring, electrocardiogram (ECG) recording, joint motion detection, and smart textiles^5,6^, as well as piezoelectric sensor arrays for electronic skin^5,6^. Regardless of the type and electrical (voltage/current) requirements of the sensors, these systems need to have sensors in the thousands to cover the appropriate areas of the body and fulfill their function.

More ambitious applications, such as haptic gloves and haptic suits or vests for AR/VR, aim at enhancing the user’s sense of touch, a complex task for which haptic feedback actuators have proven to be one of the best solutions^7,8^. The size and resolution of the actuator array are key to enhancing the user’s feeling and hence the virtual experience immersion. Typically, covering larger areas of the body requires more actuators but also results in a better experience. The driving of ultrasound transducers over extensive areas for haptic feedback systems^9,10^ is currently under exploration using TFTs.

In all of the previously addressed cases, the scaling in the size of the system quickly presents a challenge, as more functions mean more arrays covering progressively larger areas of the body to the point where, if such systems were designed with only rigid elements, they would be uncomfortable to wear due to their rigidity and weight. The scaling in sensor and actuator count also entails an increase in the power that needs to be delivered to these arrays, which can quickly reach several Watts. High output power delivery circuit blocks using TFTs are not only critical for the realization of such advanced systems but have also remained unexplored. Previous power delivery circuits in TFT technology have focused mainly on the µW–mW range^11–18^, far from the Watt-level requirements of large-area sensor and actuator arrays.

In this work, we focus on exploring the capabilities and limitations of TFT technology to enable Watt-level power delivery by means of one vital power delivery circuit block, the boost DC-DC converter. Boost converters generate an output voltage larger than their input voltage while maintaining a certain current level at their output required by the load. We specifically focus on the circuit’s performance along with the limitations, challenges and compromises involved in its design when utilizing low-temperature polysilicon (LTPS) TFT technology. In order to freely explore the technology’s limitations, closed-loop regulation blocks have been omitted in this work and will be the focus of future endeavors.

This work is organized as follows. First, the performance of a fully flexible (delaminated) diode-connected TFT boost converter is presented. Subsequently, the trade-offs between single gate (SG) and dual gate (DG) design are examined by comparing the delaminated diode-connected TFT boost converter with two other similar circuits designed with DG TFTs. Next, an additional switch-connected TFT boost converter is introduced, which achieved the highest efficiency performance amongst all versions. Later, the impact of parasitic elements on this type of TFT power delivery circuits is discussed, and the advantages of parasitic reduction are illustrated in two different ways: by means of an additional boost converter in which the external inductor is soldered directly on top of the thin-film and by careful measurements of the parasitic elements specific to the proposed designs (see “Methods” section). Finally, this paper concludes with a discussion about the most relevant results and the remaining challenges to make flexible large-area sensor and actuator systems a reality.

## Results

### Diode-connected TFT boost converter

**Fig. 2:**
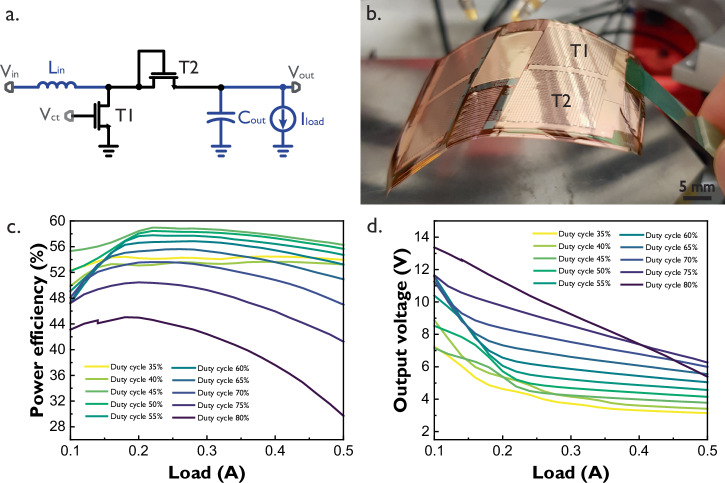
SG diode-connected boost converter. **a** Schematic. **b** Photograph of the delaminated sample. **c** Efficiency vs load curve **d** Output voltage vs. load curve. All curves were measured with a 71 kHz square signal at node *V*_ct_ of transistor T1. Input voltage is 3.3 V. Inductor L_in_ and capacitor C_out_ are external. This converter’s area is 44.5 mm × 27 mm including the flex connector footprint. Key findings: the converter’s efficiency increases with the duty cycle up to a given point (45% duty cycle) and peaks at 58.9% efficiency with a 0.22 A load. Loads up to 0.5 A can be supported with an acceptable efficiency of 56.3%. Very high output voltages and large loads incur power-efficiency trade-offs.

The diode-connected TFT boost converter is designed using LTPS TFT technology on a glass substrate, where each power transistor contains 1 million parallel 50 μm/4.5 μm SG TFTs. Previous work has demonstrated the high-output power achieved by this circuit. However, these results focused only on output power delivery and correspond to a non-delaminated version of the circuit, thus not flexible^19^. In this work, the diode-connected TFT boost converter was delaminated from the glass substrate before its characterization. A photograph, once the laser delamination process^20^ has been completed, is shown in Fig. 2b, while the converter’s efficiency and voltage performance for several duty cycle values for a square control signal applied to node *V*_ct_ are presented in Fig. 2c, d, respectively.

The efficiency of the diode-connected TFT boost converter increases along with the duty cycle of the control signal at node *V*_ct_, reaches its peak for a duty cycle of 45% and then drops due to dynamic power dissipation. The circuit achieves a maximum efficiency of 58.9% at a load of 0.22 A, with a corresponding output voltage of 4.78 V. While the step-up from the 3.3 V input may seem modest, the same configuration can support higher loads up to 0.5 A, maintaining an efficiency of 56.3% and an output voltage of 3.78 V. This heavy load configuration can become useful for resistive sensor arrays, which do not require extremely high voltages to read their sensors but do require high currents if many of the sensors are to be read in parallel. A maximum output voltage ripple of 0.8 V was measured for a 50% duty cycle and is shown in Supplementary Fig. 1 along with the inductor’s current and voltage curves. The output voltage ripple is mainly affected by the considerable current swing in the inductor and the choice of the external output capacitor.

If higher voltages are necessary, a duty cycle of 65% will ensure output voltages between 11.6 and 5.5 V while maintaining the efficiency of the circuit above 47% for loads from 0.1 to 0.5 A. The highest efficiency achieved with this 65% duty cycle configuration is 55.6% with a 0.26 A load and a corresponding output voltage of 6.87 V. Progressively higher output voltages can be achieved at the cost of efficiency or load current.

It is worth mentioning that the delimitation process will influence the performance of power TFTs in these circuits. In the case of transistor T2 in Fig. 2, an *R*_ON_ degradation of 9% was measured over a corresponding gate-source voltage *V*_GS_ = 5 V, see Table 1. In contrast, switch transistor T1 operates at voltages that do not exceed *V*_GS_ = 3.3 V, which limits its degradation. Future work should consider the optimization of the delamination process to minimize its impact on the power TFTs.

**Table 1:** *R*_ON_ degradation of power TFTs

*V*_GS_ (V)	3.3	4	5	6	7	8
*I*_DS_ On glass (μΑ)	26.1	34.62	44.12	52.46	59.87	66.51
*I*_DS_ Delaminated (μΑ)	25	32	40	46.2	51.7	56.6
Degradation (%)	4	7.5	9	13.7	13.6	14.8

Average current *I*_DS_ before and after delamination for 181 power TFTs measured across 20 different wafers.

Voltage *V*_DS_ was kept constant at 0.1 V.

The proposed diode-connected TFT boost converter requires external passive elements mounted on a PCB for testing. Such elements include an 18 μH inductor and a 220 μF capacitor. The use of an external PCB puts considerable distance in between the external passive elements and the power TFTs, this distance and the connection methods to the PCB add additional undesirable parasitic elements, mainly resistances and capacitances, that reduce power efficiency. The effects of these parasitic elements will be explored later in this work, and a possible solution will be proposed.

### Single gate vs. dual gate TFTs for high output power boost converter design

An additional advantage of TFT technology is the possibility to fabricate both SG and DG transistors on the same substrate with individual control on each gate. Typically, the back gate (BG) from DG transistors is an additional gate that can be used to further influence the flow of electrons through the transistor’s channel. This influence in the channel can, in turn, be used to shift the threshold voltage of the transistors^21,22^. If both the top gate (TG) and the BG of a TFT are used in tandem, one can increase the transconductance of the transistor, implying a potential reduction of the transistor size while maintaining approximately the same output current.

**Fig. 3:**
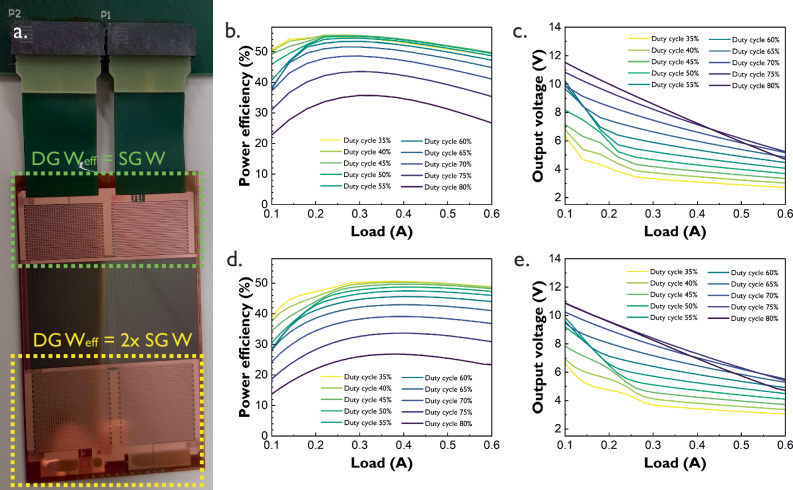
DG diode-connected boost converters. **a** (top) The effective width *W*_eff_ of the power TFTs is the same as the width from the SG delaminated boost converter presented in Fig. 2. (bottom) The effective width *W*_eff_ of the power TFTs is double the width of the SG delaminated boost converter presented in Fig. 2. **b** Efficiency vs load curve for the top boost converter 2. **c** Output voltage vs load curve for the top boost converter **d** Efficiency vs load curve for the bottom boost converter. **e** Output voltage vs load curve for the bottom boost converter. All curves were measured with a square control signal of 70.5 kHz at node *V*_ct_ of transistor T1. Input voltage is 3.3 V. Inductor L_in_ and capacitor C_out_ are external. The top converter area is 44.5 mm × 20 mm, while the bottom converter occupies 44.5 mm × 27 mm, including the flex connector footprint. Key findings: DG TFTs can be used to reduce area footprint of the proposed converters while maintaining performance, the smaller area improves device variability and parasitics since less devices are needed for a given output current.

A simple way to differentiate both boost converter designs shown in Fig. 3a is through the effective width (*W*_eff_) of their power TFTs. As a first-order approximation, a DG TFT can be considered to have a *W*_eff_ that is twice the size of the width (*W*) of a SG TFT as long as the physical dimensions of both TFTs are the same, excluding parasitics. Hence, Fig. 3a shows at the top a converter where approximately DG *W*_eff_ = SG *W* and therefore the area of the resulting circuit is reduced by 26% with respect to the SG version in Fig. 2. This size reduction already considers the footprint area of the flex connector, which itself accounts for 25% of the total area of the DG converter.

The efficiency and output voltage characteristics (Fig. 3b–e) of both converters exhibit similar trends. This confirms that the BG of DG TFTs can be used to reduce the area of certain transistors, such as power TFTs, for a given output current. Both converters achieve peak efficiency at a low duty cycle, 45% for the top converter and 35% for the bottom one. The top converter reaches a maximum efficiency of 55.4% at a load of 0.22 A, while the bottom converter achieves 50.6% at 0.38 A. At maximum efficiency, the output voltage step-up remains moderate, with 3.85 V for the top converter and 3.47 V for the bottom one. If higher output voltages are necessary, increasing the duty cycle can achieve voltages up to 10 V, though at the cost of higher power consumption. Even at 70% duty cycle for the top converter and 55% for the bottom one, efficiency remains above 40% across most of the current load range. The top converter generally outperforms the bottom one, likely due to its smaller footprint, which reduces variability between TFTs and minimizes parasitic resistive and capacitive elements. Both converters were measured while still on the glass substrate.

It is worth noting that the previously reported maximum efficiency of 54.9% at 0.4 A^19^, observed before delamination of the SG converter in the previous section, is also achieved by the smaller DG top converter in this section at a similar current load of 0.38 A. This result suggests that delamination may slightly shift the optimum operating point of this type of converter. In contrast, the DG bottom converter was unable to exceed 50% efficiency due to several factors, one of them being the additional parasitic elements introduced by the extra metal needed to connect the BGs of twice the number of transistors with respect to the top converter.

### Switch-connected TFT boost converter

**Fig. 4:**
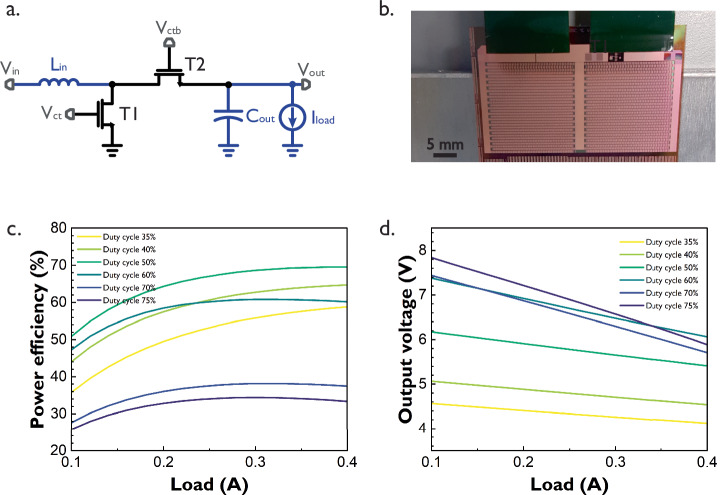
SG switch-connected TFT boost converter. **a** Schematic. **b** Photograph **c** Efficiency vs. load curve **d** Output voltage vs. load curve. All curves were measured with two out-of-phase 42 kHz control signals at nodes *V*_ct_ and *V*_ctb_ of transistors T1 and T2, respectively. Input voltage is 3.3 V. Inductor L_in_ and capacitor C_out_ are external. This converter’s area is 44.5 mm × 27 mm, including the flex connector footprint. Key findings: the switch-connected converter achieves higher efficiency and more linear output voltages w.r.t. the diode-connected converter, but will incur larger losses at high duty cycles.

Previous converters exhibit efficiencies as high as 58.9% while delivering considerable amounts of power. To improve the efficiency of this type of power delivery block using LTPS transistors, an alternative switch-connected TFT boost converter was proposed, as shown in Fig. 4.

The switch-connected TFT boost converter replaces the diode-connected transistor in previous circuits by another power transistor T2 acting as a switch. Despite the more complex driving circuit for this extra transistor, this circuit achieves higher efficiencies and more linear output voltages (Fig. 4c, d) with respect to the load compared to the previous diode-connected boost converters.

The maximum efficiency of the switch-connected TFT boost converter is 69.5% for a 0.4 A load with a corresponding output voltage of 5.4 V (duty cycle of 50%). However, increasing the duty cycle to achieve higher output voltages is not a viable strategy for this converter, as it significantly raises its dynamic power dissipation, leading to efficiency loss earlier than in the diode-based versions. This increased dynamic power dissipation results from the charge-discharge of the large parasitic capacitances of both TFT switches, as well as the energy required to drive both relatively large gates. In contrast to the diode-connected boost converter, the gate of T2 in the switch-connected version must be charged and discharged along with all its parasitic resistances and capacitances at every cycle, which incurs more losses with respect to its diode-connected counterpart. Metal line overlaps also contribute to dynamic losses, with the gate connection of T2 being a significant difference with respect to the diode-connected boost converter. An additional difference between the switch-connected and the diode-connected converters is the channel resistance of T2, which is larger in the former case than in the later, a larger resistance will dissipate more power the longer the switch is on. Moreover, increasing the load will also contribute to temperature performance degradation caused by excessive power dissipation.

A maximum output voltage ripple of 0.67 V was measured for a 60% duty cycle and is shown in Supplementary Fig. 2 along with the inductor’s voltage and the converter’s control signals. Once more, the output voltage ripple is mainly affected by the considerable current swing in the inductor and the choice of the external output capacitor.

### Parasitic elements in high output power thin-film converter design

**Fig. 5:**
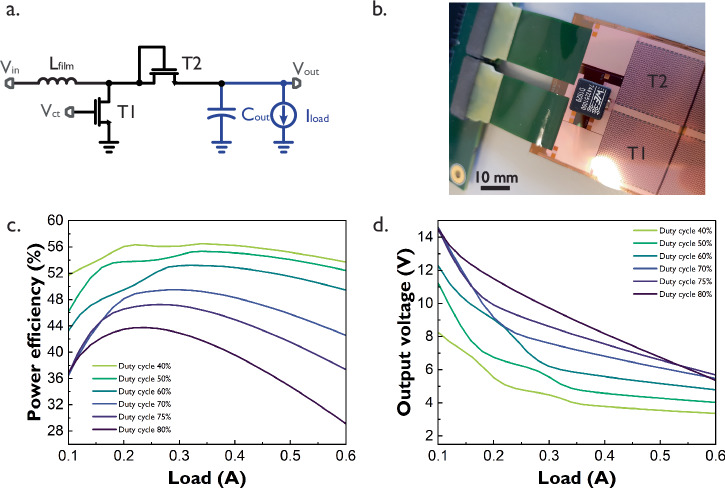
SG internal-inductor TFT boost converter. **a** Schematic **b** Photograph **c** Output voltage vs. load curve. **d** Output voltage vs. load curve. All curves were measured with a 51 kHz square signal at node *V*_ct_ of transistor T1. Input voltage is 3.3 V. Inductor L_film_ was soldered directly onto the film. Capacitor C_out_ is external. Input voltage is 3.3 V. This converter’s area is 55.3 mm × 35 mm, including the flex connector footprint.

Parasitic elements are known to significantly impact the performance of power delivery blocks. Larger parasitic elements lead to greater performance degradation in the corresponding circuit. The same phenomena hold for TFT technologies. To proof the impact of parasitics on the proposed power converters, an internal-inductor TFT boost converter has been designed, see Fig. 5. This converter consists of a diode-connected circuit similar to the one shown in Fig. 2, with the key difference that the external inductor is soldered directly onto the thin-film using a method that carefully controls the heat during the soldering process to protect the flexible film on glass (see “Methods” section). As a result, the distance between the inductor and the TFT-based converter has been strongly reduced, positively impacting the parasitic resistances and capacitances.

The performance of the internal-inductor TFT boost converter while remaining on a glass substrate is presented in Fig. 5c, d. The efficiency at a duty cycle of 40% increased considerably with respect to the diode-connected TFT boost converter in Fig. 2c, reaching a maximum value of 56.5% with a 0.38 A load and a corresponding output voltage of 4.03 V. Despite the lack of change for higher duty cycles, the efficiency at a duty cycle of 40% not only increased but also became less variable across load currents. This consistency can be seen both at lower loads (0.1 A with an efficiency of 51.3% and output voltage of 8.25 V) and at higher loads (0.5 A with an efficiency of 55.2% output voltage of 3.55 V). The output voltages of the internal-inductor TFT boost converter are higher than the diode-connected TFT boost converter ones for all duty cycles, illustrating the importance of a low parasitic resistance in power delivery blocks.

### Stress test of high output power TFT boost converters

**Fig. 6:**
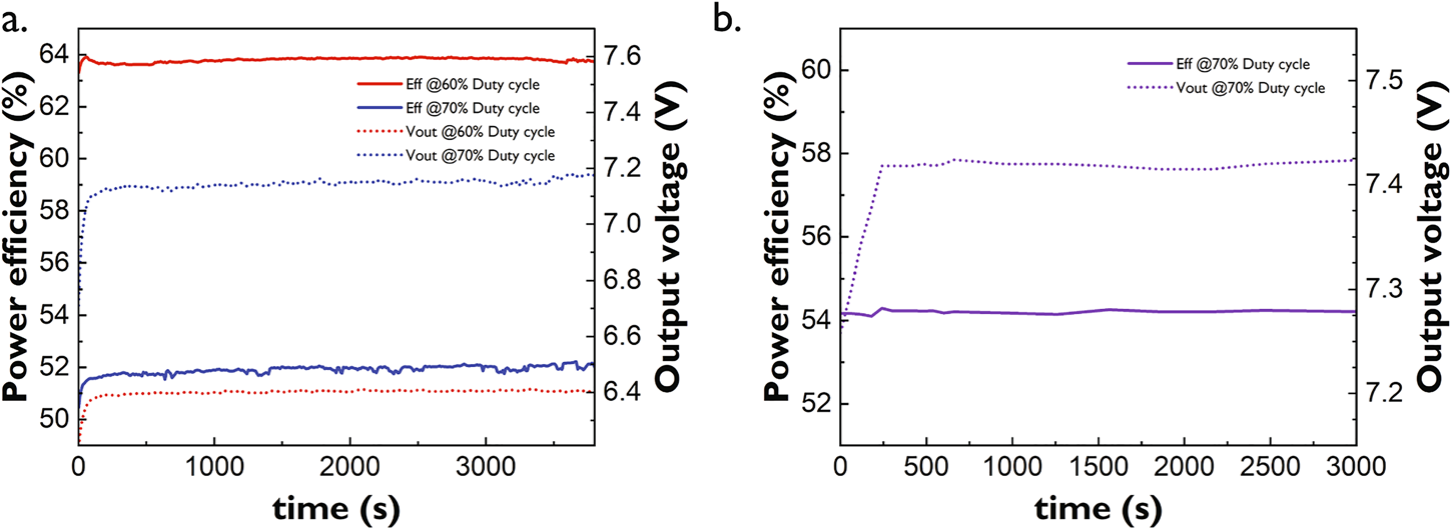
One-hour stress test results. **a** Efficiency and output voltage vs time for the switch-connected TFT boost converter. **b** Efficiency and output voltage vs. time for the diode-connected TFT boost converter. All curves were measured at a fixed load of 0.4 A. The switch-connected TFT boost converter’s control signals are 42 kHz, while the diode-connected TFT boost converter’s control signal is 57 kHz. Input voltage is 3.3 V. Inductor L_in_ and capacitor C_out_ are external. The slower ramp-up curve of the diode-connected TFT boost converter is due to an undesired delay introduced by the measurement setup.

Power delivery blocks, such as boost converters, must operate reliably over extended periods. It is therefore crucial that they maintain their performance without significant degradation over time. Figure 6 presents one-hour stress measurements for a fixed load, comparing the switch-connected TFT boost converter and the diode-connected TFT boost converter. Both converters sustain their operating point throughout the test, exhibiting minimal variations in efficiency and output voltage. However, the diode-connected TFT boost converter displays a slower ramp-up curve compared to the switch-connected version, attributed to an unintended delay in the measurement setup.

A maximum voltage variation of the diode-connected TFT boost converter of 2.28% is obtained, including the ramp-up section, while the switch-connected TFT boost converter yields voltage variations of 3.31 and 6.97% for duty cycles of 60 and 70%, respectively. Excluding the steep ramp-up of the switch-connected TFT boost converter at 70% duty cycle results in a reduced voltage variation of 3.49%.

In terms of efficiency, both converters exhibit acceptable variability. The diode-connected TFT boost converter shows a minimal variation of only 0.23%, while the switch-connected TFT boost converter varies by 0.96 and 3.48% for duty cycles of 60 and 70%, respectively. However, when excluding the steep ramp-up phase of the switch-connected TFT boost converter at 70% duty cycle, the efficiency variation is reduced to 1.29%.

## Discussion

One of the key research fields for TFT electronics is the realization of large-area electronic sensor and actuator arrays, whereby the research towards power delivery circuits is unexplored. The diversity of targeted applications implies that it is considerably difficult to cover all cases with only one power delivery circuit. Nevertheless, in this work, we explored the capabilities and limitations of LTPS TFT technology in high output power delivery circuits, focusing specifically on the most important trade-offs in the design of boost converters.

The first implementation was the diode-connected TFT boost converter, which performed most optimal at lower loads (around 0.2 A–0.3 A) or at higher output voltages (11.6 V), resulting in acceptable efficiencies (>47%). The switch-connected TFT boost converter topology delivered the highest efficiencies (69.5%) at very high loads (0.4 A) but yielded moderate output voltages (slightly less than double its input voltage). TFT platforms employing DG TFTs can significantly reduce the footprint of power TFTs within LTPS technologies. The effects of parasitic elements were reduced by developing a method to solder discrete surface-mount chips directly into the thin-film. The manner in which these elements affect the converter's performance and measurements is further explored in the Methods section of this work. Finally, the stability of this technology was demonstrated throughout one-hour stress tests, where the diode-connected TFT boost converter showed maximum output voltage and efficiency variations of 2.28 and 0.23%, while the switch-connected TFT boost converter showed maximum output voltage and efficiency variations of 3.31 and 0.96%, respectively.

Table 2 compares the proposed converters with state-of-the-art designs in thin-film transistor technologies and demonstrates that prior art operates orders of magnitude below the power delivery levels demonstrated in this work. Most prior work has focused on display-related circuits, where load current levels are significantly lower, and area constraints are far more stringent. Our proposed converters are designed to deliver high power values to large-area systems, which are significantly larger than most displays. Our targeted systems incorporate numerous sensors and actuators, resulting in load currents that are orders of magnitude larger due to the significant number of active elements. The proposed thin-film converters achieve very high output power delivery while maintaining efficiencies of up to 69.8%.

**Table 2:** Comparison of this work to state-of-the-art solutions in thin-film technology

Ref.	^11^	^12^	^13^	^14^	^15^	^16^	^17^	^18^	This work		
Result type	Sim.	Sim.	Sim.	Meas.	Meas.	Meas.	Sim.	Meas.	Measurement		
Tech.	a-IGZO				P-only LTPS	a-Si:H	LTPS	LTPO	LTPS		
DCDC type	Charge pump	Cross C.	Cross C.	Cross C.	Diode & Cross C.	Charge pump	Charge pump	Cross C.	Diode boost	SW boost	Ind. boost
W/L (µm/µm)	320/20	320/20	2500/10	-	-	3000/5	-	200/6 (a-IGZO) 20/6 (LTPS)	50,000,000/4.5		
Cout (nF)	0.15^c^	0.2	1	0.5	1	0.01^c^	-	0.012	220 k		
Ind. (µH)	-	-	-	-	-	-	-	-	18		
Vin (V)	3	8	5	10	5	5, 8	5	3	3.3		
Vout (V)	5.8	12.7, 15.2	16.6	20.8	8.8	13, 20	9	14.5	11.6^d^, 4.78^e^, 5.43^f^	6.17^g^, 5.4^g^	8.25^h^, 4.03^h^
Vout ripple (V)	0.75	-	-	-	0.0045	-	-	0.6	0.8^g^	0.67^f^	NA^i^
Iload (A)	5 µ	~150 µ, ~175 µ	550 µ	250 µ	250 µ	-	30 µ	90 µ	0.1^d^, 0.22^e^, 0.4^f^	0.1^g^, 0.4^g^	0.1^h^, 0.38^h^
Total load regulation (V/A)	60k^j^	60 k^j^, 167 k^j^	20 k^j^	9.25 k^j,a^	3.3 k^j^	-	12.5 k^j^	36.5 k^j^	13.65	2.56	9.78
Eff. (%)	91.1	60.7, 90	52	66.6	79.6	-	90	83.3^a^	47^d^, 58.9^e^, 55.4^f^	50.9^g^, 69.5^g^	51.3^h^, 56.5^h^
Control signal freq. (Hz)	1 M	200 k	1 M	1 M^a^	1 M	60 k	20 k	500 k	71 k	42 k	51 k
Area (mm^2^)	-	-	0.58^k^	14.8^b^	4.76^b^	-	-	0.45^k^	1201.5 (SG), 890 (DG)	1201.5	1935.5 (Ind. incl.)
Year	2021	2020	2019	2010	2008	2006	2005	2023	2025		

^a^Simulation result

^b^Area including TFT and internal capacitors

^c^Pumping capacitances, no data about output capacitance

^d^65% Duty cycle

^e^45% Duty cycle

^f^60% Duty cycle

^g^50% Duty cycle

^h^40% duty cycle

^i^Due to limitations during measurements

^j^Estimation from output voltage figures

^k^TFT area only (capacitor excluded).

Even though no closed-loop regulation was implemented in this work, an important performance metric for DC–DC converters, such as load regulation (expressed as ∆*V*_out_ /∆*I*_load_) can still be derived. One must be careful interpreting these results as they are obtained while testing the technology’s limitations and thus under extreme load variations, which may be atypical from application-specific designs. The large load current variations convert into considerable output voltage changes due to various factors such as the absence of a closed-loop regulation block, the large parasitic elements resulting from the size of the TFTs and their routing and the mobility limitations of TFTs compared to their CMOS counterparts. External factors such as the choice of the output capacitor can also influence this metric. The load regulation metric in Table 2 was calculated using the data corresponding to the highest efficiency case for all boost converters. The difference between this work and prior art in the TFT domain stems from the difference of several orders of magnitude in load currents.

As the power levels of the converters approach the Watt range, it becomes essential to compare these results with silicon CMOS solutions. To facilitate this comparison, two commercial chips and two academic references^23^^,24^ with similar specifications were selected. Table 3 compares this work with the previously mentioned silicon CMOS solutions and shows that TFT technology is capable of Watt-level power delivery with comparable metrics compared to CMOS chips, including power efficiency, output voltage, output voltage ripple and load regulation. The performance differences between traditional CMOS and LTPS lead to the necessity of larger power transistors for LTPS to limit performance degradation. For large-area applications, the ability to conform to surfaces may be more critical than area constraints, making thin-film transistor-based implementations particularly advantageous. In addition, the efficiency of the switch-connected TFT boost converter is comparable to the commercial silicon chips, while all three of the proposed TFT converters exhibit a higher efficiency compared to the 0.18 µm BCD converter^23^. Comparisons have been made carefully, as the performance of converter circuits is highly dependent on their application. Nevertheless, Table 3 provides context for the performance levels of boost converters in LTPS technology, highlighting their trade-offs.

**Table 3:** Comparison of this work to commercial silicon CMOS chips

Part number	^23^	^24^	RV-3.305S/R6.4	RV-3.305S/P	This work		
Output power (W)	1.2	1	2	2	1.05 – 2.17	0.62–2.16	0.83–1.53
Tech.	0.18 µm BCD	140 nm CMOS	CMOS	CMOS	LTPS		
DCDC type	Cross C.	Switched Cap.	-	-	Diode boost	SW boost	Ind.boost
Cout (µF)	10	10^d^	-	-	220		
Ind. (µH)	-	-	-	-	18		
Vin (V)	4–5.5	2.6–4.2	3–3.6	3–3.6	3.3		
Vout (V)	5	5	5	5	11.6^h^, 4.78^i^, 5.43^j^	6.17^k^, 5.4^k^	8.25^l^, 4.03^l^
Vout ripple (V)	0.030	0.050<	0.2	0.2	0.8^k^	0.67^j^	NA^m^
Iload (A)	0.24^e^	0.2^c^	0.4	0.4	0.1^h^, 0.22^i^, 0.4^j^	0.1^k^, 0.4^k^	0.1^l^, 0.38^l^
Total load regulation(V/A)	-	-	2.08	2.08	13.65	2.56	9.78
Eff. (%)	51	94.5	80	75	47^h^, 58.9^i^, 55.4^j^	50.9^k^, 69.5^k^	51.3^l^, 56.5^l^
Control signal freq. (Hz)	90 M	4 M	50 k	50 k	71 k	42 k	51 k
Area (mm^2^)	120	8.1^a^	475.5^b^	475.5^b^	1201.5 (SG), 890 (DG)	1201.5	1935.5 (Ind. incl.)
Thickness (mm)	~1.4 mm^f^	0.5	11.1^g^	11.1^g^	0.020		
Year	2022	2015	-	-	2025		

*BCD* Bipolar-CMOS-DMOS

Chips found through the Digikey website: https://www.digikey.be

^a^PCB area

^b^Full chip package

^c^Manual calculation from paper data for 1 W of output power

^d^An additional 10 µF input capacitor, as well as 4 additional 100 nF floating capacitors are required

^e^Manual calculation from paper data for 1.2 W of output power

^f^Approximation from LGA package datasheets

^g^Includes through hole pins

^h^65% Duty cycle

^i^45% Duty cycle

^j^60% Duty cycle

^k^50% Duty cycle

^l^40% Duty cycle

^m^Due to limitations during measurements.

It must be acknowledged that the ability of TFT technology to bend and conform to different surfaces may also be an additional source of stress with respect to CMOS solutions. Future work will have to address the effects in performance caused by long-term bending cycles as well as flexibility under mechanical load. Effective temperature dissipation must also be explored if these types of circuits are to be in touch or close to the human body. Supplementary Fig. 3 shows infrared camera captures of the proposed boost converters, illustrating that careful circuit layout is key to avoid too many hot spots in said converters. Additionally, application-dependent blocks such as closed-loop regulation circuits must also be studied and implemented in order to derive more in-depth performance metrics, such as line regulation, on LTPS TFT technology in the context of large output power delivery. Closed-loop regulation can be challenging due to the mobility limitations of TFTs compared to CMOS. Further exploration of different regulation techniques is required to verify whether the design of these loops directly on film yields adequate results. Hybrid solutions, such as soldering a CMOS feedback controller onto the film for closed-loop regulation, may be an acceptable first step towards achieving proper closed-loop performance metrics prior to their implementation directly on film.

In essence, this research work examines the unexplored realm of high output power DC-DC boost converter design in LTPS thin-film technology. Essential design trade-offs such as circuit topology, transistor size, SG and DG device utilization and parasitic elements have been discussed by means of four TFT boost converters. The proposed TFT boost converters obtain efficiencies between 47 and 69.5% and deliver output power values starting at 0.62 W and up to 2.17 W. These results are comparable to commercial CMOS circuits and close the gap with more advanced academic CMOS solutions, see Table 3. This work enables the realization of flexible large-area sensor and actuator arrays in LTPS technology, where output power delivery and physical constraints, such as rigidity, thickness, and weight, can precede CMOS technology’s advantages, such as small chip surface area.

## Methods

### Testing of high-output power TFT converters

As previously mentioned, parasitic elements affect the performance of power delivery circuits and must be taken into consideration during testing. Supplementary Fig. 4a, b shows efficiency and output voltage performance curves for the previously presented diode-connected and switch-connected TFT boost converters. The difference is the method used for testing. On one hand, for the switch-connected TFT boost converter, a comparison is drawn between measurements performed using flex connectors to connect the power TFTs to an external PCB for testing and utilizing spring-loaded pins (pogo pins) in the PCB, which can land directly on top of the glass at designated spots. On the other hand, for the diode-connected TFT boost converter, a comparison is made between measurements using flex connectors while the sample is still attached to the glass substrate and measurements performed after delamination.

Results from the switch-connected converter demonstrate that the general trend of both the efficiency and the output voltage curve is the same. However, flex connectors result in higher efficiency, but slightly lower output voltages compared to the pogo pins. These results indicate that different testing methods will affect the converter’s performance figures. In this case, impedance measurements (performed with the HM8118 LCR bridge from Rhode & Schwarz) indicate that the parasitic resistance of an individual flex connector (*Z*_flex_ = 0.199 + j0.112) is larger than that of a pogo pin (*Z*_pogo_ = 0.004 + j0.077) for a frequency of 55 KHz. The increased parasitic resistance of the flex connector reduces the output voltage of the circuit. However, when looking at the efficiency, the flex connector circuit appears to have better results. This can be explained by the way in which these measurements are obtained. During efficiency measurements, the input voltage is fixed by means of a voltage supply (DC power supply E3613A from Keysight) while the output current is maintained by the converter itself. The increased parasitic resistance of the flex connectors present at the input of the converter reduces the current that feeds the actual circuit. If the input current is further reduced by other layout parasitic elements (input line and output line layouts at the flex level are not perfectly symmetrical), the overall circuit might appear to have an increase in efficiency compared to the pogo pin variant. Flex connectors were chosen for all measurements since they are essential if the circuits are meant to be delaminated and flexible.

Regarding the diode-connected TFT boost converter, the general trend of both the efficiency and the output voltage curve is the same for both the on-glass and the delaminated versions. Minimal differences appear in the efficiency curve, and a lower output voltage can be seen for the delaminated case. This confirms that, as previously stated, delamination can physically affect the samples, but the process will not result in catastrophic damage.

Parasitic resistances were optimized in both the PCB and the flex connector design, and 4-point resistance measurements were performed for various nodes of the circuit (4156 C parametric analyzer from Agilent). Each node was measured starting at the external PCB and up to the thin-film. This measured the PCB parasitic resistance from the PCB lines and the flex connectors. Even after this optimization, most resistances sit between 0.11 Ω to 0.3 Ω, which can still considerably affect performance with load currents as high as the ones handled by the proposed converters. Moreover, this parasitic resistance for each connection point (input, output and ground) is at least 15% of the *R*_ON_ resistance value (<0.95 Ω) of the switch TFTs as reported earlier^19^. Assuming an input current of 3 A, a drop of 0.4 V can be easily introduced before the inductor and at the connection to ground.

### Soldering of the external inductor directly onto the thin-film

Soldering was performed using SnAgCu305-type 5 solder paste, which was dispensed manually onto the solder pads using a 25GA needle. The inductor was placed on the substrate using a flip-chip bonder, as the pads on the bottom of the component were not visible to place it manually. The whole assembly was soldered in a Vapor Phase Reflow Oven using a linear solder profile with a peak temperature of 245 °C. In this type of reflow oven, the transfer of the heat to the solder is done via the vapor of a pure vapor phase oil, which conducts much better than air and therefore, soldering can be done at a lower temperature without the worries of overheating, thus protecting the film.

## Supplementary information

41528_2026_536_MOESM1_ESM.

## Data Availability

No datasets were generated or analyzed in this study. All data corresponding to experimental results generated or analyzed during this study are included in this published article and its supplementary information file.

## References

[1] Geng, D. et al. Thin-film transistors for large-area electronics. *Nat. Electron* **6**, 963–972 (2023).

[2] Fu, B., Pu, C., Guo, L., Xu, H. & Peng, C. A Wearable ultrasound transducer array for neuromodulation applications in the treatment of diabetic foot disease. In *Proc*. *IEEE International Ultrasonics Symposium, IUS*, 10.1109/IUS51837.2023.10308018 (2023).

[3] Sadeghpour, S., Ingram, M., Wang, C., D’Hooge, J. & Kraft, M. A 128× 1 Phased array piezoelectric micromachined ultrasound transducer (pmut) for medical imaging. In *Proc. 21st International Conference on Solid-State Sensors, Actuators and Microsystems, TRANSDUCERS 2021*, 34–37, 10.1109/TRANSDUCERS50396.2021.9495521 (2021).

[4] Pelgrims, J., Myny, K. & Dehaene, W. A 36V Ultrasonic driver for haptic feedback using advanced charge recycling achieving 0.20CV2f power consumption. In *Proc.**ESSCIRC 2021**-**IEEE 47th European Solid State Circuits Conference, Proceedings*, 159–162, 10.1109/ESSCIRC53450.2021.9567765 (2021).

[5] Pang, C. et al. A flexible and highly sensitive strain-gauge sensor using reversible interlocking of nanofibres. *Nat. Mater.* **11**, 795–801 (2012). 22842511 10.1038/nmat3380

[6] Khalid, M. A. U. & Chang, S. H. Flexible strain sensors for wearable applications fabricated using novel functional nanocomposites: a review. *Compos. Struct.* **284**, 115214 (2022).

[7] Yang, Y. et al. Touch the metaverse: demonstration of haptic feedback in network-assisted augmented reality. In *Proc*. *2024 IEEE International Conference on Pervasive Computing and Communications Workshops and other Affiliated Events (PerCom Workshops*), 379–381, 10.1109/PERCOMWORKSHOPS59983.2024.10503143 (2024).

[8] Cai, S. et al. ViboPneumo: a vibratory-pneumatic finger-worn haptic device for altering perceived texture roughness in mixed reality. *IEEE Trans. Vis. Comput. Graph* 1–14, 10.1109/TVCG.2024.3391877 (2024). 10.1109/TVCG.2024.3391877 38648153

[9] Pelgrims, J., Myny, K. & Dehaene, W. An ultrasonic driver array in metal-oxide thin-film technology using a hybrid TFT-Si DLL locking architecture. *IEEE J. Solid State Circuits* **59**, 516–527 (2024).

[10] Pelgrims, J., Myny, K. & Dehaene, W. A low power dynamic circuit topology towards a-IGZO thin-film ultrasonic transducer driving circuit. In *Proc*. *FLEPS 2021**-**IEEE International Conference on Flexible and Printable Sensors and Systems,*10.1109/FLEPS51544.2021.9469863 (2021).

[11] Sharma, A., Bahubalindruni, P. G., Bharti, M. & Barquinha, P. On-chip power supply generation for self-contained electronics using oxide thin-film transistors. *Int. J. Circuit Theory Appl.* **49**, 2112–2121 (2021).

[12] Tiwari, B., Bahubalindruni, P. G., Goes, J. & Barquinha, P. Positive-negative DC-DC converter using amorphous-InGaZnO TFTs. *Int. J. Circuit Theory Appl.* **48**, 394–405 (2020).

[13] Lei, T., Liao, C., Huang, J., Yang, J. & Zhang, S. P-108: oxide thin film transistors integrated DC-DC converter with high efficiency for passive RFID tag. *SID Symp. Dig. Tech. Pap.* **50**, 1660–1663 (2019).

[14] Hong, S. H. et al. DC-DC converters using indium gallium zinc oxide thin film transistors for mobile display applications. *Jpn J. Appl. Phys.* **49**, 03CB05 (2010).

[15] Yoon, J.-S., Kang, J.-S. & Kwon, O.-K. 38.2: highly efficient P-type only cross-coupled DC-DC converter using low temperature poly-Si (LTPS) TFTs for mobile display applications. *SID Symp. Dig. Tech. Pap.* **39**, 545–548 (2008).

[16] Choi, J. W. et al. Stability of DC-DC converter using hydrogenated amorphous thin-film transistors. *J. Korean Phys. Soc.* **48**, 98 (2006).

[17] Yeh, S.-H., Sun, W.-T., Chen, C.-C. & Yang, C.-S. 43.4: a novel integrated DC-DC converter using LTPS TFT. *SID Symp. Dig. Tech. Pap.* **36**, 1442–1445 (2005).

[18] Priyadarshi, S., Rahaman, A., Billah, M. M., Nahar, S. & Jang, J. A compact DC-DC converter using low-temperature poly-Si oxide thin-film transistors. *IEEE Electron Device Lett.* **44**, 1640–1643 (2023).

[19] Papadopoulos, N., Lopez, M. V., Ameys, M., Huang, T. C. & Myny, K. 11-4: 3.55-watt output power LTPS TFT DCDC converter for actuators on wearable devices on flexible substrate. *SID Symp. Dig. Tech. Pap.* **54**, 132–135 (2023).

[20] Coulson, P. et al. A 3,072-electrode multiplexed µECoG array for high-density and large-area cortical recordings based on scalable thin-film electronics. in *Bioel2024* (Johannes Kepler University Linz, 2024).

[21] Liu, X., Shi, R., Jiang, W., Xie, X. & Wong, M. Comparator with non-uniform parameter compensation using dual-gate thin-film transistors. *IEEE Electron Device Lett.*10.1109/LED.2024.3359597 (2024).

[22] Chen, Y., Nie, C., Han, B. & Zhang, S. High Vth compensation range AMOLED pixel circuit based on a dual gate a-IGZO TFT and a specially amplified capacitor. In *Proc*. *2023 6th International Conference on Electronics Technology, ICET**2023*, 396–400, 10.1109/ICET58434.2023.10211860 (2023).

[23] Pan, D. et al. A 1.2W 51%-Peak-Effi ciency Isolated DC-DC Converter with a Cross-Coupled Shoot-Through-Free Class-D Oscillator Meeting the CISPR-32 Class-B EMI Standard. *2022 IEEE International Solid-State CircuitsConference (ISSCC)*, pp. 240–242, (San Francisco, CA, USA, 2022)

[24] Piqué, G. V., Bergveld, H. J. & Karadi, R. A 1W 8-ratio switched-capacitor boost power converter in 140nm CMOS with 94.5% effi ciency, 0.5mm thickness and 8.1mm 2 PCB area. *Symposium on VLSI Circuits (VLSI Circuits)*, pp. C338–C339, (Kyoto, Japan, 2015).

