# Supplementary material for: High output power low temperature polysilicon thin-film transistor boost converters for large-area sensor and actuator applications
Source: Npj Flex Electron. 2026 Jan 27;10(1):32. doi: 10.1038/s41528-026-00536-6 (PMC12948667; doi:10.1038/s41528-026-00536-6)
Supplement: Supplementary file 1 — 41528_2026_536_MOESM1_ESM. [file 41528_2026_536_MOESM1_ESM.pdf]

High output power Low Temperature Polysilicon Thin-Film Transistor boost converters for large area sensor and actuator applications

Supplementary Materials

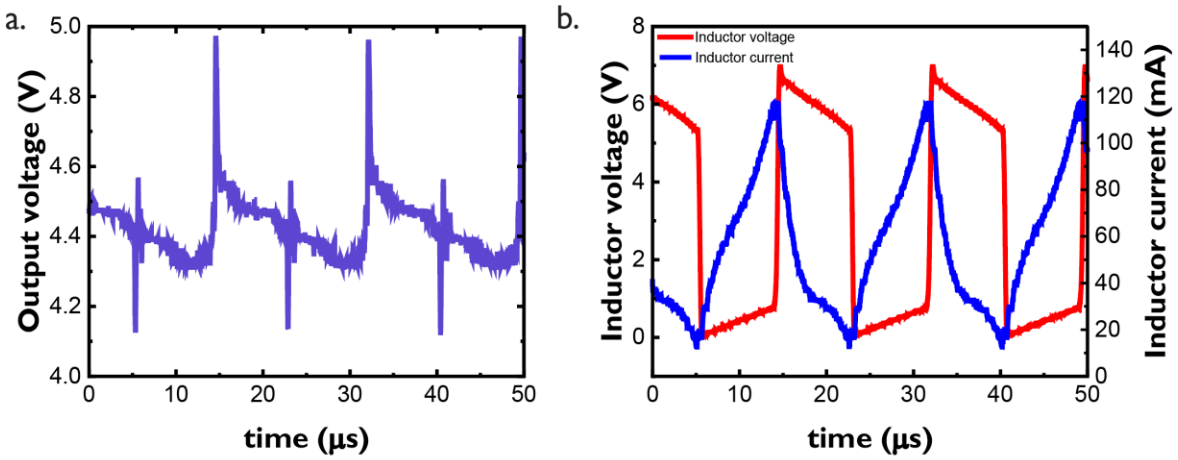

Supplementary Fig. 1. Steady-state curves of the diode-connected boost converter for a 50% duty cycle. **a.** Output voltage. **b.** Inductor current and inductor voltage (at the drain of T1). The output voltage ripple is 0.8 V in this particular case.

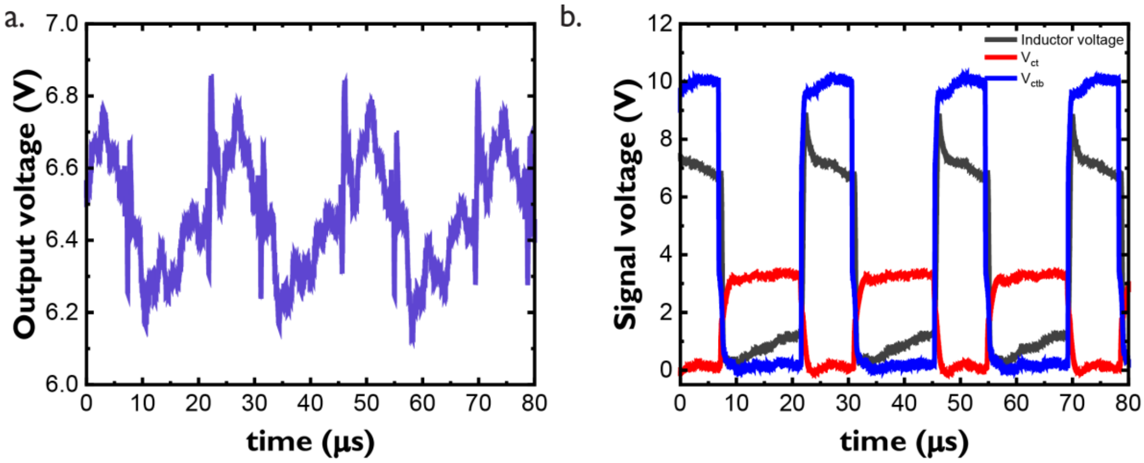

Supplementary Fig. 2. Steady-state curves of the switch-connected boost converter for a 60% duty cycle. **a.** Output voltage. **b.** Inductor current and inductor voltage (at the drain of T1). The output voltage ripple is 0.67 V in this particular case.

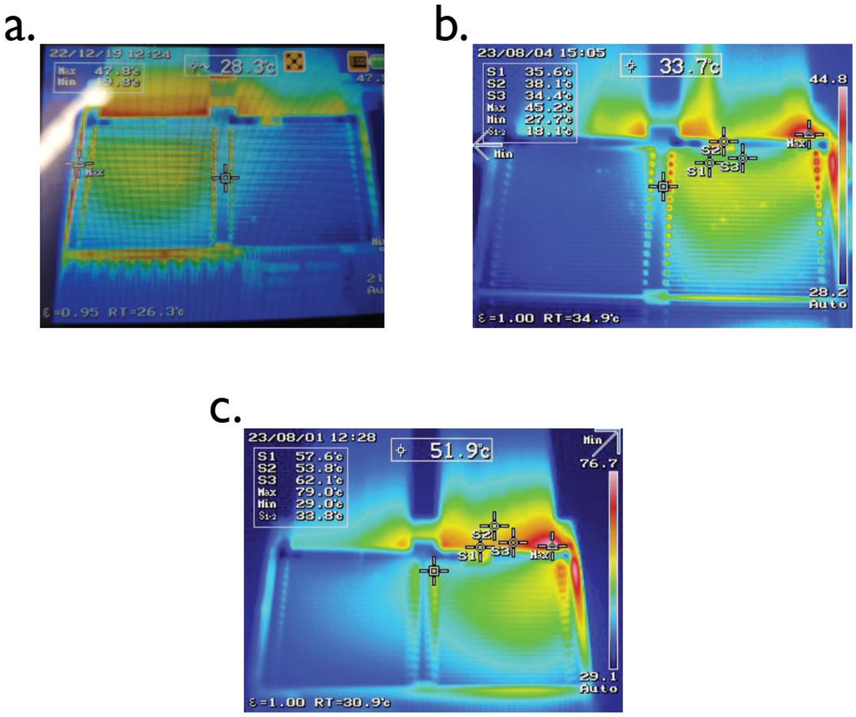

Supplementary Fig. 3. Temperature measurements of the proposed boost converters. **a.** Diode-connected boost converter. Input voltage is 3.3V with a 57 kHz square signal and 50% duty cycle and 0.4 A load. **b.** Large DG diode-connected boost converter. Input voltage is 3.3V with a 52 kHz square signal and 50% duty cycle and 0.4 A load. **c.** Switch-connected boost converter. Input voltage is 3.3V with a 42 kHz square signal and 70% duty cycle and 0.4 A load.

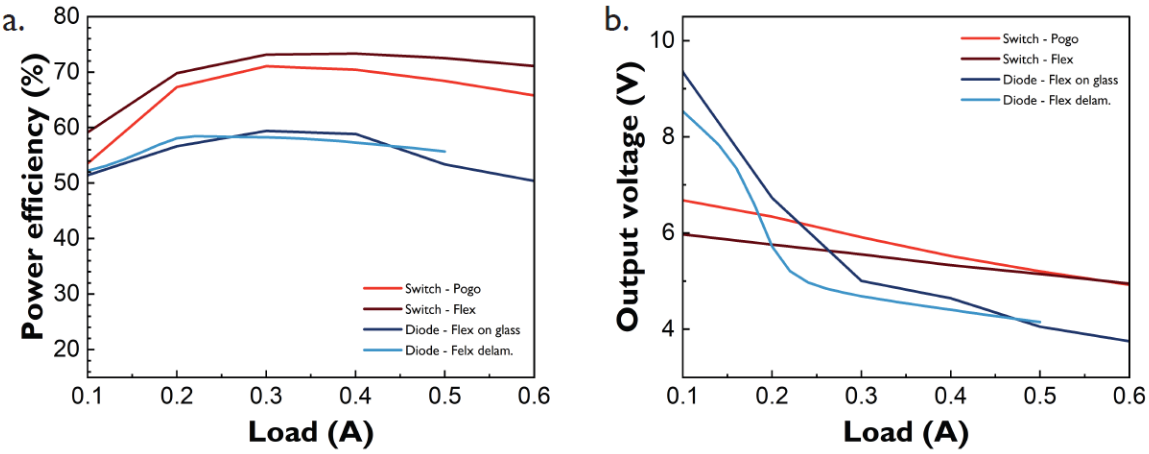

Supplementary Fig. 4. Impact of parasitic elements introduced by different measurement methods. **a.** Efficiency vs load curve for different measurement methods for switch-connected and diode-connected TFT boost converters. **b.** Output voltage vs load curve for different measurement methods for switch-connected and diode-connected TFT boost converters. The control signals for the switch-connected TFT boost converter are the same as in Fig. 4 while the one for the diode-connected TFT boost converter is the same as in Fig. 2. Input voltage is 3.3V for all converters.
